# Perceived Stress and Short-Form Video Application Addiction: A Moderated Mediation Model

**DOI:** 10.3389/fpsyg.2021.747656

**Published:** 2021-12-23

**Authors:** Yinbo Liu, Xiaoli Ni, Gengfeng Niu

**Affiliations:** ^1^School of Humanities and Social Sciences, Xi’an Jiaotong University, Xi’an, China; ^2^School of Psychology, Central China Normal University, Wuhan, China

**Keywords:** perceived stress, short-form video applications, self-compensation motivation, shyness, moderated mediation model

## Abstract

Nowadays, short-form video applications have become increasingly popular due to their strong appeal to people, especially among college students. With this trend, the phenomenon of short-form video application addiction (SVA) also become prominent, which is a great risk for individuals’ health and adaptation. Against this background, the present study aimed to examine the association between perceived stress and SVA addiction, as well as its mechanism—the mediating role of self-compensation motivation (SCM) and the moderating role of shyness. A total of 896 Chinese college students was recruited to complete a set of questionnaires on perceived stress (PS), SCM, shyness, and short-form video applications. The results show that PS was positively associated with SVA, and SCM partially mediated this association. In addition, both the direct association between PS and SVA and the indirect effect of SCM were moderated by shyness and were stronger for individuals with higher levels of shyness. The results could not only deepen our understanding of the underlying factors of SVA but also provide suggestions for relevant prevention and intervention procedures.

## Introduction

Researchers have conducted valuable work in the past on the negative effects of excessive use of online apps and electronic devices (i.e., smartphones, online games, social network sites) on users. Additionally, it has been well established that problematic use poses real threats to users’ mental health, sleep quality, and interpersonal relationships (Liu et al., 2018; Dobbs et al., 2020).

A short-format video application is a type of social media application that enables users to create and upload short-form videos and share their wonderful life moments. Nowadays, short-form video applications have achieved popularity worldwide. In China, the number of users of short-form video applications has exceeded 870 million, and the usage time accounts for 8.8% of all types of apps, including online games (China Internet Network Information Center, 2020, 2021). Short-form video applications are especially useful for sending customized content based on an analysis of user preferences; users can also choose to watch the specific creators and content in which they are most interested. This personalization mechanism allows short videos to continuously provide users with content that meets their preferences; however, it also increases the possibility of addiction (Zhang et al., 2019). Research suggests that short-form video application addiction (SVA) may be another subcategory of Internet addiction and could cause negative influences on individuals’ adaptation and well-being (Zhang et al., 2019). Due to the popularity of these applications, SVA has been a prominent social problem. Previous studies have found that, as a key risk factor for individuals’ development and adaptation, perceived stress is positively associated with SVA, However, the underlying mechanism is still unclear. In particular, college students, being the main users of Internet applications, are at high risk of Internet application addiction (Li H. et al., 2016; Deng et al., 2017; Tian et al., 2019). Therefore, examinations of college students’ SVA are of great theoretical and practical significance. Against this background, we aimed to examine the association between perceived stress and short-form video application addiction, as well as its mechanisms—the mediating role of self-compensation motivation (SCM) and the moderating role of shyness.

### Perceived Stress and Short-Form Video Applications Addiction

Perceived stress (PS) is defined as a situation appraised as threatening or otherwise demanding, wherein no sufficient resources are available for coping with the situation (Cohen et al., 1983). PS has been widely found to be closely related to various negative outcomes, such as behavioral problems (smartphone addiction) and emotional problems (anxiety, anger, and depression) (Gökçearslan et al., 2018; Zheng et al., 2019). However, when demographic variables and other psychological characteristics are controlled, stress is still the only important and significant predictor of negative outcomes (Kardefelt-Winther, 2014). Furthermore, PS may influence cognitive processes and, thereby, cause attention to shift to short-term rewards (Brand et al., 2016); thus, PS affects the way people engage in addictive disorders (Canale et al., 2019).

In the current information era, using specific Internet applications can be considered a way to relieve stress (Young and de Abreu, 2011; Snodgrass et al., 2014); PS is also found to be strongly associated with the excessive or problematic use of smart devices and Internet applications (Chen et al., 2017; Gökçearslan et al., 2018; Liu et al., 2018; Hou et al., 2019). Specifically, studies have revealed that PS is closely related to Internet-relevant problematic behaviors, and individuals who perceive more stress are more likely to be addicted to the Internet (Gao F. et al., 2018; Liu et al., 2018) and smartphones (Chung and Kim, 2010; Chiu, 2014; Cheng and Hong, 2017; Gao T. et al., 2018; Hou et al., 2019; Busch and Mccarthy, 2021). Regarding short-form video applications, they can provide users with lighthearted content to relieve stress anytime and anywhere. This behavior of obtaining short-term rewards has become increasingly widespread among users (Brand et al., 2016). Unfortunately, this popularity provides unprecedented opportunities for the development and dissemination of more short-form video applications and may further lead to SVA at a young age (Dubey et al., 2020). At the same time, studies have focused mainly on the direct effect of PS, while research on the specific influence mechanism is relatively lacking (Liu et al., 2018; Hou et al., 2019). To better explore the relationship between PS and SVA, we examined the mediating effect of SCM and the moderating effect of shyness.

### Self-Compensation Motivation as a Mediator

Self-compensation motivation refers to the motivation that causes individuals to take certain actions to meet their psychological needs and compensate for their dissatisfaction with reality when they are hurt or threatened (Chu et al., 2020a). When individuals tend to meet their compensation needs online, it may lead to the problem of Internet overuse (Chu et al., 2020b). Especially, according to the compensatory Internet use model, for eliminating or relieving the stress caused in real life, as individuals often tend to use the Internet as a means of self-regulation and compensation. This process may yield two results: the positive result is that individuals compensate for their missing needs in real life with the help of the Internet; the negative result is that they rely on the Internet for compensation and overuse it, leading to the emergence of addiction symptoms (Kardefelt-Winther, 2014). In other words, users can temporarily escape from the negative impacts of real-life stress through the use of short-form video applications (Wang et al., 2015; Chu et al., 2020b). However, if their reliance on the Internet for compensation leads to its overuse, this behavior may increase their risk of SVA. Considering the popularity of smart devices, especially college students tend to use the functions of entertainment and relaxation provided by short-form video applications to deal with the stress of the real world. This process may alleviate the negative emotions brought about by stress, but this coping style may also lead to SVA.

Motivation is an important factor that affects users’ behavior when using Internet products. When individuals are stressed in real life, certain motivations may lead to a risk of problematic outcomes (Wang et al., 2015). Individuals are more likely to suffer from self-attrition when encountered with stress (Baumeister et al., 2007); by making them tend to compensate for themselves (Kardefelt-Winther, 2014; Jin et al., 2017). Therefore, we believe that PS can improve individuals’ motivation to seek self-compensation, and driven by SCM, individuals are more likely to have unhealthy Internet use behaviors, including SVA.

Based on this information, it was hypothesized that:

Hypothesis 1: PS and SCM are positively associated with SVA.
Hypothesis 2: SCM may mediate the relationship between PS and short-form video addiction.

### Shyness as a Moderator

Shyness refers to that people feel nervous and uncomfortable when they encounter a new social environment or respond to social evaluation. Henderson and Zimbardo (2001) defined shyness as a state of discomfort in a social environment, usually accompanied by behavioral inhibition or frustration, which greatly affects an individual’s ability to achieve goals or willingness to actively participate in social activities. According to the general theory of addiction (Jacobs, 1986), the potential factors for the interaction between stress and addiction behaviors are personality variables. There are individual differences in the association between stress and addiction behavior, shyness may be one of them (Caplan, 2002; Chak and Leung, 2004; Gao T. et al., 2018). Shy individuals tend to show more social withdrawal and adjustment problems (Wang et al., 2020). When faced with real-life stress, shy individuals prefer to seek support from online social networks rather than offline (Chan and Cheng, 2016; Chen et al., 2017). Compared with reality, individuals with high levels of shyness would feel more relaxed when dealing with people and things in virtual space, which means that they are more inclined to seek ways to deal with stress from the Internet. This may cause problems in their Internet use behavior. In fact, many researchers have confirmed that shyness can be an important predictor of internet and mobile phone addiction (Chak and Leung, 2004; Ayas, 2012; Han et al., 2017; Tian et al., 2017). Individuals with high levels of shyness may be more likely to have addictive behavior when they use the Internet (i.e., short-form video applications) to cope with PS. In addition, the typical manifestation of shyness is a lack of social skills (Iranmanesh et al., 2019). According to the cognitive-behavioral model of pathological Internet use (Davis, 2001), in real life, individuals who do not have good interpersonal interaction tend to hold negative beliefs, for example, they lack social skills in face-to-face interaction. Their self-presentation ability in online environments is better than that during face-to-face interaction, so they have higher motivation to use the Internet (Caplan, 2005). Shy people also are more willing to maintain social relationships in the network environment (Ebeling-Witte et al., 2007; Clayton et al., 2013) and use the Internet as an important means of entertainment (Yu et al., 2019). However, this may weaken their interaction in real life (Gao T. et al., 2018), resulting in a lack of social resources and support (Porter and Chambless, 2017), and failure to obtain the internal resources to cope with the stress. However, according to previous research and social compensation models, shy individuals tend to get social resources and support from online activities such as social networks or online games (Li X. et al., 2016; Sioni et al., 2017; Miao et al., 2018). So shy individuals are more inclined to compensate for the lack of reality through online activities, and the stimulation brought by the network is more effective for the satisfaction of SCM. In other words, individuals with a high level of shyness tend to seek comfort and compensation from the online world driven by SCM, rather than seeking relief and help in the real world. Unfortunately, once shy individuals obtain the social resources and support they need from the Internet, they will spend more time online (Tian et al., 2017; Gao T. et al., 2018). Namely, shy individuals may be more likely to engage in problematic usage behaviors.

In conclusion, individuals with high levels of shyness are more likely to use online products to cope with real stress and develop SVA. In addition, individuals with a high level of shyness are more likely to use web applications as a channel for coping with stress driven by SCM, which may further lead to SVA. It was further hypothesized that:

Hypothesis 3: The link between PS and SVA may be moderated by shyness.
Hypothesis 4: The link between SCM and SVA may be moderated by shyness.

## Materials and Methods

### Participants and Procedure

We originally recruited 1,010 participants aged 18–22 years (SD = 0.88, M = 19.76) from two colleges in Shandong Province, East China, using stratified cluster sampling. All participants answered the questionnaire in their classrooms; they were told that the data collected were for research purposes only and that all information would remain anonymous and confidential. Participants were informed of the voluntary principle of the study. No participants opted out, but 17 participants were excluded for missing data relating to the main variables; another three participants and were excluded because they had no experience with any short-form video applications. Thus, a total of 990 college students completed our survey, and 52.2% of the participants were female. This study was approved by the Ethics Committee of Psychological Research of the authors’ institution.

### Measurements

#### Perceived Stress

In this study, the Perceived Stress Scale Chinese Version (S) was used. The CPSS was used to measure the degree of an individual’s subjective stress in life and their perception of their ability to cope with stressful events. The CPSS has 14 self-assessment items and uses a 5-point Likert scale (0 = never; 4 = very often). The higher the score, the greater the PS. This scale demonstrated high reliability and validity in a sample of Chinese college students (Ge et al., 2019). The Cronbach’s alpha coefficient for the scale was 0.885.

#### Self-Compensation Motivation

The SCM scale compiled by Jin et al. (2017) was adapted to measure. According to the definition of self-compensation, three questions were proposed to measure the motivation for self-compensation. In this study, we replaced the description of “consumption” in the original scale with “use short-form video application” and revised the description of items appropriately: (1) When facing stressful situations, using short-form video applications will make me feel better. (2) Using short-form video applications will make me make up for the disappointments in real life. (3) Using short-form video applications can compensate me psychologically. Participants scored answers using a 7-point Likert scale (1 = totally disagree, 7 = totally agree) and we aggregated the scores for each question to form the index of SCM. The Cronbach’s alpha coefficient for the scale was 0.842.

#### Shyness

The revised Henderson Undergraduate Shyness Scale (RHUSS) was used in this study, which was adapted from the Henderson and Zimbardo Shyness Scale (Henderson and Zimbardo, 2001), translated and revised by Wang et al. (2009). The RHUSS has 17 items and consists of four aspects: need for approval, self-accusation, fear of refusal, and inhibition of self-expression. This scale uses a 5-point Likert scale (1 = strongly disagree; 5 = strongly agree). The higher the total score, the higher the shyness level. The scale has good reliability and validity for the Chinese population (Han et al., 2017). The Cronbach’s alpha coefficient for the scale was 0.813.

#### Short-Form Video Application Addiction

In this study, we used the item adapted by Zhang et al. (2019) to measure SVA. The questionnaire consists of six items that are scored using a 7-point Likert scale (1 = totally disagree, 7 = totally agree), and we aggregated the scores for each question to form the index of SVA. This questionnaire has good reliability and validity among Chinese college students (Zhang et al., 2019). The Cronbach’s alpha coefficient for the scale was 0.899.

### Statistical Analysis

In this study, SPSS 22.0 and PROCESS macro 24.0 were used for data analysis. Firstly, The Harman single factor test was used to determine common method bias, and then the Pearson’s correlation coefficient was used for the correlational analysis of all the variables. At last, the PROCESS macro was adopted to assess the estimators and 95% confidence intervals for direct and indirect effects with 5,000 bootstrap samples.

## Results

### Description of Statistics

Pearson correlations, as well as the means and standard deviations of the main variables, were presented in Table 1. As expected, PS was positively correlated with SVA (*r* = 0.422, *p* < 0.001) and SCM (*r* = 0.158, *p* < 0.001). Additionally, SCM was positively correlated with SVA (*r* = 0.343, *p* < 0.001), and shyness was positively correlated with both PS (*r* = 0.241, *p* < 0.001) and SVA (*r* = 0.274, *p* < 0.001).

**TABLE 1:** Descriptive statistics and correlation matrix of all variables.

Variables		*M*	*SD*	1	2	3	4
1	PS	1.435	0.294	–			
2	SVA	1.271	1.271	0.422***	–		
3	SCM	1.056	1.056	0.158***	0.343***	–	
4	Shyness	0.769	0.770	0.241***	0.274***	0.057	–

N = 990. ***p < 0.001. PS, perceived stress; SCM, self-compensation motivation; SVA, short-form video application addiction.

### Testing for the Mediating Model

**FIGURE 1:**
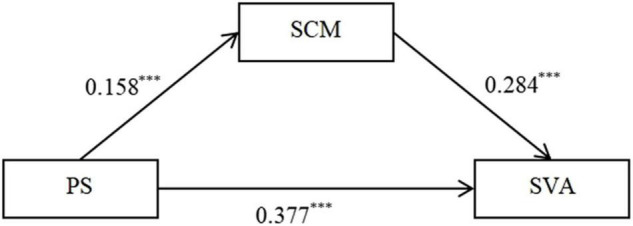
The mediation model, all coefficients standardized. PS, perceived stress; SCM, Self compensation motivation; SVA, short-form video application addiction. ***p < 0.001.

In this study, PROCESS macro v3.0 for SPSS (Model 4) was adopted to test the total effect of PS on SVA and the mediating effect of SCM. The total effect of PS on SVA indicated that PS was positively associated with SVA (total effect = 0.422, *p* < 0.001, 95% CI = 0.347–0.473); this result supported Hypothesis 1. SCM was then added to the model. As shown in Figure 1, PS was significantly and positively predicted SCM (β = 0.158, *t* = 5.024, *p* < 0.001, CI = 0.096–0.220). SCM can indirectly and significantly predict SVA (β = 0.284, *t* = 10.207, *p* < 0.001, CI = 0.229–0.338). At the same time, the direct effect of PS on SVA was significant (β = 0.377, *t* = 15.573, *p* < 0.001, CI = 0.323–0.432). Therefore, SCM played a partial mediating role in the association between PS and SVA (indirect effect = 0.045, 95% CI = 0.027–0.063), supporting Hypothesis 2.

### Moderated Mediating Model Analysis

Shyness was anticipated to moderate the direct association between PS and SVA, as well as the second stage of the indirect relationships in Hypotheses 3 and 4. The two hypotheses were tested by estimating a moderated mediation model (Model 15) with PROCESS macro (Hayes, 2013).

**FIGURE 2:**
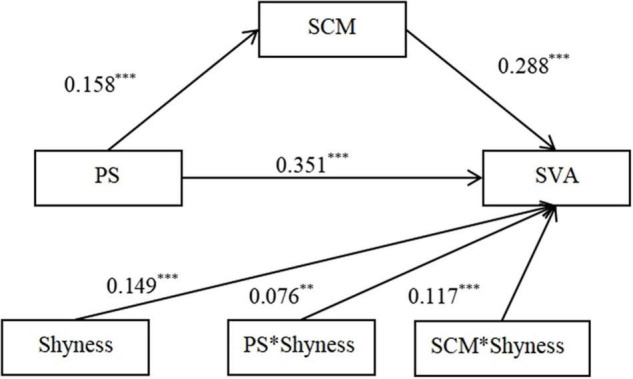
The moderated mediation model, all coefficients standardized. PS, perceived stress; SCM, Self compensation motivation; SVA, short-form video application addiction. **p < 0.01 and ***p < 0.001.

**FIGURE 3:**
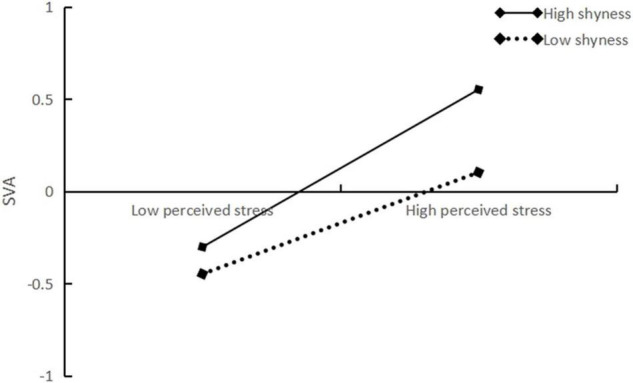
Plot of the relationship between perceived between stress and SVA at two levels of shyness.

**FIGURE 4:**
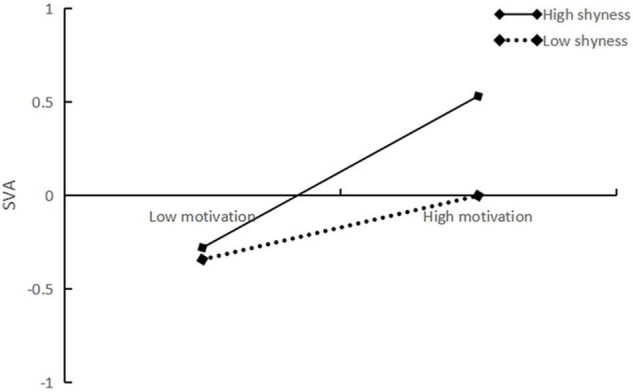
Plot of the relationship between SCM and SVA at two levels of shyness.

The results indicated that shyness was significantly and positively predicted with SVA (β = 0.149, *t* = 5.235, *p* < 0.001, CI = 0.093–0.205). In addition, the direct association between PS and SVA was significant (β = 0.351, *t* = 12.540, *p* < 0.001, CI = 0.296–0.406), and this path was moderated by shyness (β = 0.076, *t* = 2.554, *p* < 0.01, CI = 0.018–0.134) (Figure 2). Similarly, the association between SCM and SVA was significant (β = 0.288, *t* = 10.718, *p* < 0.001, CI = 0.235–0.341), and this path was also moderated by shyness (β = 0.117, *t* = 3.952, *p* < 0.001, CI = 0.059–0.176). We plotted the results for SVA predicted by PS and SCM separately for low (one standard deviation below the mean) and high (one standard deviation above the mean) shyness. Simple slope tests revealed that with the increase in the level of shyness, PS prediction effect on SVA gradually expanded (Low shyness: β = 0.283, *t* = 8.011, *p* < 0.001, CI = 0.214–0.352; High shyness: β = 0.433, *t* = 9.410, *p* < 0.001, CI = 0.343– 0.524). This supports Hypothesis 3 (see Figure 3). SCM’s positive predictive effect on SVA gradually rose along with the increase in the level of shyness (Low shyness: β = 0.183, *t* = 4.974, *p* < 0.001, CI = 0.111–0.255; High shyness: β = 0.416, *t* = 9.620, *p* < 0.001, CI = 0.331– 0.501), supporting Hypothesis 4 (see Figure 4). In addition, both direct and indirect effects were significant, regardless of the level of shyness (Table 2).

**TABLE 2:** Conditional indirect effects of PS on SVA through shyness ± 1 SD (*N* = 990).

Shyness	PS					
	Direct			Indirect		
	Effect	SE	95%CI	Effect	SE	95%CI
Low	0.275	0.037	0.202–0.348	0.027	0.009	0.013–0.045
Moderate	0.351	0.028	0.296–0.406	0.046	0.001	0.027–0.067
High	0.427	0.044	0.340–0.513	0.064	0.014	0.040–0.093

## Discussion

With the popularity of mobile network technology and smart devices, the addiction to these Internet or online applications has increased (Gökçearslan et al., 2018). Especially, the popularity of short-form video applications and smart devices leads to dependence on smartphones, which may be reflected by the overuse of short-form video apps.

The aim of this study was to investigate the effects of PS on SVA and the related internal mechanism. Correlation analysis revealed a significant positive relationship between PS and SVA. By further constructing a moderated mediation model, we found that PS positively predicted SVA both directly and indirectly *via* SCM; thus, SCM emerged as a significant partial mediator in the association between PS and SVA. Additionally, the moderating effect of shyness was also confirmed. Specifically, shyness promoted both the effect of PS on SVA and the effect of SCM on SVA.

### The Direct Effect of Perceived Stress and the Mediating Role of Self-Compensation Motivation

The main motivation behind addiction is the desire to escape the stress and negative emotions of real-life (Stetina et al., 2011). The effect of PS on addiction has garnered considerable empirical support. However, there is still a lack of relevant discussion concerning short-form videos. As we expected, there was a positive association between PS and SVA, which is consistent with previous studies on Internet addiction. Among the influencing factors, stress is the most prominent. Hence, when examining the relationship between psychological factors and Internet addiction, the influence of stress should be considered (Kardefelt-Winther, 2014). The general strain theory also points out that stress is the main factor contributing to problem behaviors (Agnew, 2001; Jun and Choi, 2015). To deal with stress in life, individuals may choose passive coping styles, such as spending a lot of time on the Internet to avoid problems (Chen et al., 2017). In other words, addiction may have become a way to deal with stress (Chiu, 2014). Furthermore, this study found that SCM could mediate the association between PS and SVA. PS can lead to a lack of sufficient resources to cope with the difficulties or requirements encountered. Individuals consume limited internal resources to cope with stress (Wang et al., 2015; Zheng et al., 2019). According to the strength model of self-control (Baumeister et al., 2007; Meldrum et al., 2015) and the limited resource model of self-control (Muraven and Baumeister, 2000; Han et al., 2017; Geng et al., 2018), the consumption of internal resources will cause ego depletion, which will further lead to a series of behavioral problems (Baumeister et al., 2007). This may make individuals inclined to seek self-compensation (Kardefelt-Winther, 2014). However, uncontrolled self-compensation can lead to out-of-control behaviors such as substance abuse, bulimia, and Internet addiction (Baumeister et al., 2007; Chu et al., 2020a). Kardefelt-Winther (2014) pointed out that theories that consider psychological characteristics and motivation will provide further insights into the compensatory nature of the Internet. According to the compensatory Internet use model, motivation for use is based on social and psychological problems or unsatisfactory real-life needs (Kardefelt-Winther, 2014). The negative results of PS can be alleviated by using short-form video applications, which further motivates individuals to use them. SVA can be formed and consolidated under the effect of this motivation. The mediating model of this study reveals such a mechanism: individuals will produce SCM under the effect of PS, which may lead to the emergence of SVA.

### The Moderating Role of Shyness

In addition, in this study, we also further examined an individual difference (shyness) in the association between PS and SVA. In research on Internet addiction, shyness is regarded as a personality factor of Internet addiction (Han et al., 2017). Regarding shyness, on the one hand, shyness moderated the direct relationship between PS and SVA. This finding supports our hypothesis. Specifically, with the increase in shyness level, the effect of PS on SVA is increasingly significant. In other words, shyness magnifies the promoting effect of PS on SVA. As a negative influence from the external environment, PS leads to negative cognition and emotions. Unfortunately, shy people are more prone to more interpersonal interaction problems and more vulnerable to negative emotions (such as loneliness, depression, and anxiety). To avoid the discomfort brought about by face-to-face communication and the real world, individuals with high levels of shyness tend to integrate into the network environment to avoid these negative experiences (Han et al., 2017; Wang et al., 2020). In this case, it is possible to cause shy people to become addicted to short-form videos.

On the other hand, shyness moderated the association between SCM and SVA. As hypothesized, PS can promote SCM and further lead to SVA. However, shy individuals tend to have lower cognitive flexibility and a higher risk of cognitive failure (Yu et al., 2019), which may lead to the exaggeration of negative social effects and negative emotions, which is crucial for the development of Internet addiction, which may lead to individuals with high levels of shyness to use short-form videos as a way to participate in network activities to alleviate the negative impact of stress under the promotion of SCM. Therefore, the amplification effect of shyness is reflected through not only the direct role of PS but also the intermediary role of SCM. Therefore, shyness is a risk factor that enhances the impact of PS on the SVA of Chinese college students.

### Limitations and Implications

This study has several limitations. First, we employed a cross-sectional design; thus, no conclusions can be drawn regarding causality. However, future longitudinal studies can further examine the relationships between the relevant variables. Second, in addition to the variables involved in this study, future studies should also consider other risk factors and individual differences to further clarify the relevant factors and mechanisms of short video application addiction. Researchers could further subdivide the sources of PS, for example, separating PS from real-world situations (academic performance, intimate relationships) from that related to virtual situations (upward social comparisons and information overload in online social activities), to further explore the internal mechanisms of PS influencing SVA. Finally, the results of this research suggest that an intervention study for SVA is warranted.

Despite these limitations, this study is beneficial to the literature on the relationship between PS and smart device application addiction. At the same time, it also provides a reference for subsequent studies on the formation mechanism of short-form video applications addiction. This study theoretically reveals the influence of PS on SVA and its internal mechanism, which helps expand our understanding of the factors causing SVA and broadens the research on Internet addiction. The results of this study also have some practical implications. For example, intervention for SVA can be carried out by reducing individual stress and guiding individuals to relieve stress and meet their needs through offline channels. In this process, special attention must be paid to shy individuals.

## Conclusion

This study tested a mediation model to examine the relationship between PS and SVA. The results show that in a sample of Chinese college students, PS is a direct risk factor that leads to SVA, and SVA can be predicted by the mediating effect of SCM. Shyness moderates the direct effect of PS and the latter part of the mediating effect of SCM. These findings will help us to better understand the causes of SVA and propose control measures.

## Data Availability Statement

The raw data supporting the conclusions of this article will be made available by the authors, without undue reservation.

## Ethics Statement

The studies involving human participants were reviewed and approved by Xi’an Jiaotong University. The patients/participants provided their written informed consent to participate in this study.

## Author Contributions

YL: writing—original draft and investigation. XN: conceptualization and methodology. GN: writing—review and editing. All authors read and approved the final manuscript.

## Conflict of Interest

The authors declare that the research was conducted in the absence of any commercial or financial relationships that could be construed as a potential conflict of interest.

## Publisher’s Note

All claims expressed in this article are solely those of the authors and do not necessarily represent those of their affiliated organizations, or those of the publisher, the editors and the reviewers. Any product that may be evaluated in this article, or claim that may be made by its manufacturer, is not guaranteed or endorsed by the publisher.
